# Applications of large language models in cardiovascular disease: a systematic review

**DOI:** 10.1093/ehjdh/ztaf028

**Published:** 2025-04-01

**Authors:** José Ferreira Santos, Ricardo Ladeiras-Lopes, Francisca Leite, Hélder Dores

**Affiliations:** Cardiology Department, Setúbal, Hospital da Luz Setúbal, Luz Saúde, Estrada Nacional 10, Km 37, 2900-722 Setúbal, Portugal; Católica Medical School, Sintra Campus, Estrada Octávio Pato, 2635-631 Rio de Mouro, Lisboa, Portugal; Department of Surgery and Physiology, Faculty of Medicine of the University of Porto, Cardiovascular Research and Development Centre-UnIC@RISE, Porto, Portugal; Cardiology Department, Hospital da Luz Guimarães, Luz Saúde, Guimarães, Portugal; Católica Medical School, Sintra Campus, Estrada Octávio Pato, 2635-631 Rio de Mouro, Lisboa, Portugal; Hospital da Luz Learning Health, Luz Saúde, Lisboa, Portugal; CHRC, NOVA Medical School, Lisboa, Portugal; NOVA Medical School, Lisboa, Portugal; Cardiology Department, Hospital da Luz Lisboa, Luz Saúde, Lisboa, Portugal; CoLAB TRIALS, Évora, Portugal

**Keywords:** Large language models (LLMs), Cardiovascular disease, Prevention, Patient education, Clinical decision, Artificial intelligence

## Abstract

Cardiovascular disease (CVD) remains the leading cause of morbidity and mortality worldwide. Large language models (LLMs) offer potential solutions for enhancing patient education and supporting clinical decision-making. This study aimed to evaluate LLMs’ applications in CVD and explore their current implementation, from prevention to treatment. Following the Preferred Reporting Items for Systematic Reviews and Meta-Analyses guidelines, this systematic review assessed LLM applications in CVD. A comprehensive PubMed search identified relevant studies. The review prioritized pragmatic and practical applications of LLMs. Key applications, benefits, and limitations of LLMs in CVD prevention were summarized. Thirty-five observational studies met the eligibility criteria. Of these, 54% addressed primary prevention and risk factor management, while 46% focused on established CVD. Commercial LLMs were evaluated in all but one study, with 91% (32 studies) assessing ChatGPT. The LLM applications were categorized as follows: 72% addressed patient education, 17% clinical decision support, and 11% both. In 68% of studies, the primary objective was to evaluate LLMs’ performance in answering frequently asked patient questions, with results indicating accurate, comprehensive, and generally safe responses. However, occasional misinformation and hallucinated references were noted. Additional applications included patient guidance on CVD, first aid, and lifestyle recommendations. Large language models were assessed for medical questions, diagnostic support, and treatment recommendations in clinical decision support. Large language models hold significant potential in CVD prevention and treatment. Evidence supports their potential as an alternative source of information for addressing patients’ questions about common CVD. However, further validation is needed for their application in individualized care, from diagnosis to treatment.

## Graphical Abstract

**Graphical Abstract:**
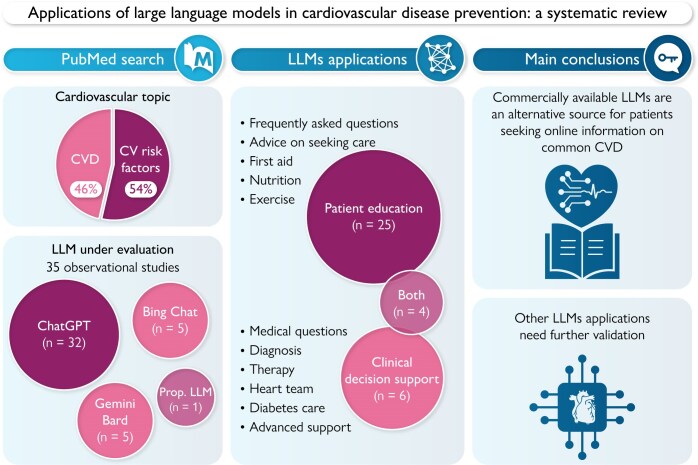

## Introduction

### Understanding large language models: a brief overview

On 30 November 2022, OpenAI launched ChatGPT, a large language model (LLM) that interacts with users in natural, everyday language. Initially released as a prototype to gather user feedback, ChatGPT rapidly gained traction, attracting over one million users within its first week.^1^ This marked the moment for public recognition and adoption of LLMs, signalling their potential to revolutionize various fields, including healthcare.

Large language models are sophisticated artificial intelligence systems engineered to understand and generate human language. Fundamentally, they function through next-word prediction based on contextual information from preceding text, enabling them to produce coherent and contextually appropriate responses. Currently, most LLMs are based on the Transformer architecture, which relies on a mechanism called self-attention. This mechanism allows the model to evaluate the importance of different words in a sentence relative to one another, effectively capturing contextual relationships and dependencies across entire sequences. The Transformer architecture consists of multiple layers of encoders and decoders, which process input data in parallel, significantly enhancing the model’s efficiency when handling large volumes of textual data.^2,3^

Large language models are trained on vast data sets to learn general language features, followed by fine-tuning on specific tasks to optimize performance in targeted domains. This training process involves adjusting the model’s parameters to minimize prediction errors, enabling the generation of contextually relevant and grammatically accurate text. Furthermore, LLMs are being developed with multimodal capabilities, allowing them to process not only text but also images, video, and audio, thereby enhancing their versatility across several applications.^2–4^

Originally designed for text generation, LLMs have demonstrated capabilities beyond basic word prediction, excelling in tasks such as language translation, summarization, and even creative writing with reasoning abilities. These capabilities have extended their applicability across numerous fields, fundamentally transforming how we interact with and manage large data sets. In medicine, LLMs hold promise by enabling the analysis of vast volumes of medical literature, patient records, and clinical research, synthesizing data to provide critical insights for healthcare professionals. This capacity is pivotal in addressing the complexities of healthcare data management, enhancing diagnostic accuracy, streamlining administrative processes, supporting personalized medicine, facilitating medical education, and advancing research, among many other applications. Large language models thus represent a transformative tool in healthcare, offering new avenues for improving patient outcomes and operational efficiency.^5–7^

### Cardiovascular disease: can large language models bridge the gaps in prevention and treatment?

Cardiovascular disease (CVD) remains the worldwide leading cause of morbidity and mortality, with approximately 70% of cases attributed to modifiable risk factors.^8^ Although effective preventive strategies, such as risk stratification tools, lifestyle modifications, and pharmacological interventions, can potentially prevent up to one in three cardiovascular (CV) events, the correct implementation and adoption of these tools remain challenging.^9^ As a striking example, in Europe, one in three physicians does not perform CV risk stratification on a regular basis during outpatient visits, often due to time constraints and cumbersome equations.^10^ Findings from the EUROASPIRE V survey revealed that 19% of patients were smokers, 38% were obese, and 66% were physically inactive. Despite medication use, 42% of patients had uncontrolled blood pressure, 71% had suboptimal cholesterol levels, and 29% had diabetes, with 46% of diabetic patients having uncontrolled HbA1c levels. These results underscore the urgent need for enhanced preventive and management CV programmes.^11^

Large language models are at the forefront of addressing these challenges with their ability to analyse vast amounts of data, including personal health records, consultation notes, exam reports, and clinical guidelines. Their computational capacity, far surpassing human capabilities, can be harnessed to develop novel strategies for patient education, clinical decision support, administrative efficiency, and data collection in cardiology-focused research.^6^

Some of the proposed future applications of LLMs in cardiology include the following:

Clinical support systems: LLMs can be employed in pre-consultation workups, automated transcription of consultations into clinical notes, generating clinical summaries, ordering diagnostic tests, ensuring guideline adherence, diagnostic support, and providing personalized risk assessment tools. Additional applications include automation of administrative tasks and enhancing medical education for healthcare professionals.^6,12–15^

Patient support systems: LLMs can enhance patient care through chatbots, tailored health reminders, symptom checkers, follow-up on patient-reported outcomes, and monitoring data from wearable devices. They also provide educational programmes and resources while facilitating communication between patients and healthcare providers.^6,14–16^

Research and development: LLMs have the capability of analysing unstructured data from electronic health records, clinical notes, and patient feedback and can identify trends, gaps, and novel associations. They can synthesize medical literature, predict patient responses to therapies, and suggest new treatment pathways and biomarkers. Additionally, LLMs can streamline clinical trial recruitment by efficiently identifying suitable patients.^2,3,6^

These promising applications highlight the potential of LLMs to bridge existing gaps in the prevention, diagnosis, and treatment of CVD by enabling the development of tools that improve risk stratification, optimize lifestyle interventions, support diagnosis, and personalize treatment plans while considering clinical guidelines and individual patient needs. This, in turn, could lead to improved health outcomes and reduced healthcare costs.

However, despite their potential, there is a critical need for rigorous scientific validation of LLMs in the context of CVD prevention and treatment. The purpose of this review was to evaluate the applications of LLMs in CVD and to explore their current implementation, from prevention to treatment. Specifically, we aimed to assess their role in clinical decision-making, patient education, risk prediction, lifestyle interventions, adherence to preventive measures, and treatment recommendations.

## Methods

This review followed the Preferred Reporting Items for Systematic Reviews and Meta-Analyses (PRISMA) guidelines, with the protocol registered in the PROSPERO database (CRD42024596678).^17^ The inclusion criteria comprised studies involving human subjects in the context of CVD prevention or treatment. Eligible studies evaluating the use of LLMs in CVD, specifically in areas such as clinical decision support, patient education, risk prediction, or lifestyle interventions, were included. Studies comparing LLM applications to standard of care or alternative interventions in CVD prevention were also contemplated. Relevant outcomes included improvements in risk factor control, patient engagement, adherence to preventive measures, diagnostic accuracy, and treatment effectiveness. The study designs considered were randomized controlled trials, observational studies, and research letters, while review articles, opinion pieces, case reports, non-peer-reviewed literature, and pre-prints were excluded. Only studies published in English within the last five years were eligible. The review focused exclusively on clinical applications of LLMs. Studies aiming at the technical development of LLMs, those using natural language processing without a specific LLM architecture, and studies involving simple chatbots not powered by LLMs were excluded from this systematic review.

A comprehensive search was conducted in PubMed to identify relevant studies on 20 October 2024. The search strategy was designed to capture relevant literature and included general keywords related to LLMs and CVD, as well as terms associated with CV risk factors and prevention (see Supplementary material online, *Table S1*). A two-stage screening process was employed to identify eligible studies. Initially, two independent reviewers (J.F.S. and H.D.) screened titles and abstracts based on the predefined eligibility criteria. Studies that met the criteria or had uncertain relevance were further subjected to full-text screening by the same reviewers. Any disagreements during the selection process were resolved through discussion and consultation with a third reviewer (R.L.-L.).

The same two reviewers independently extracted data from the selected studies using a standardized data extraction form, including study design, sample size, specific LLM applications, and key outcomes related to CVD prevention and treatment. Discrepancies were resolved by discussion. The quality of the included studies was assessed using the proper Joanna Briggs Institute (JBI) critical appraisal checklist tool, according to study design and reported as having ‘low’, ‘moderate’, or ‘high’ bias.^18^ A PRISMA flow diagram was used to document the study selection process. The key applications, benefits, and limitations of LLMs in CVD prevention and treatment were summarized.

## Results

### Study selection

**Figure 1:**
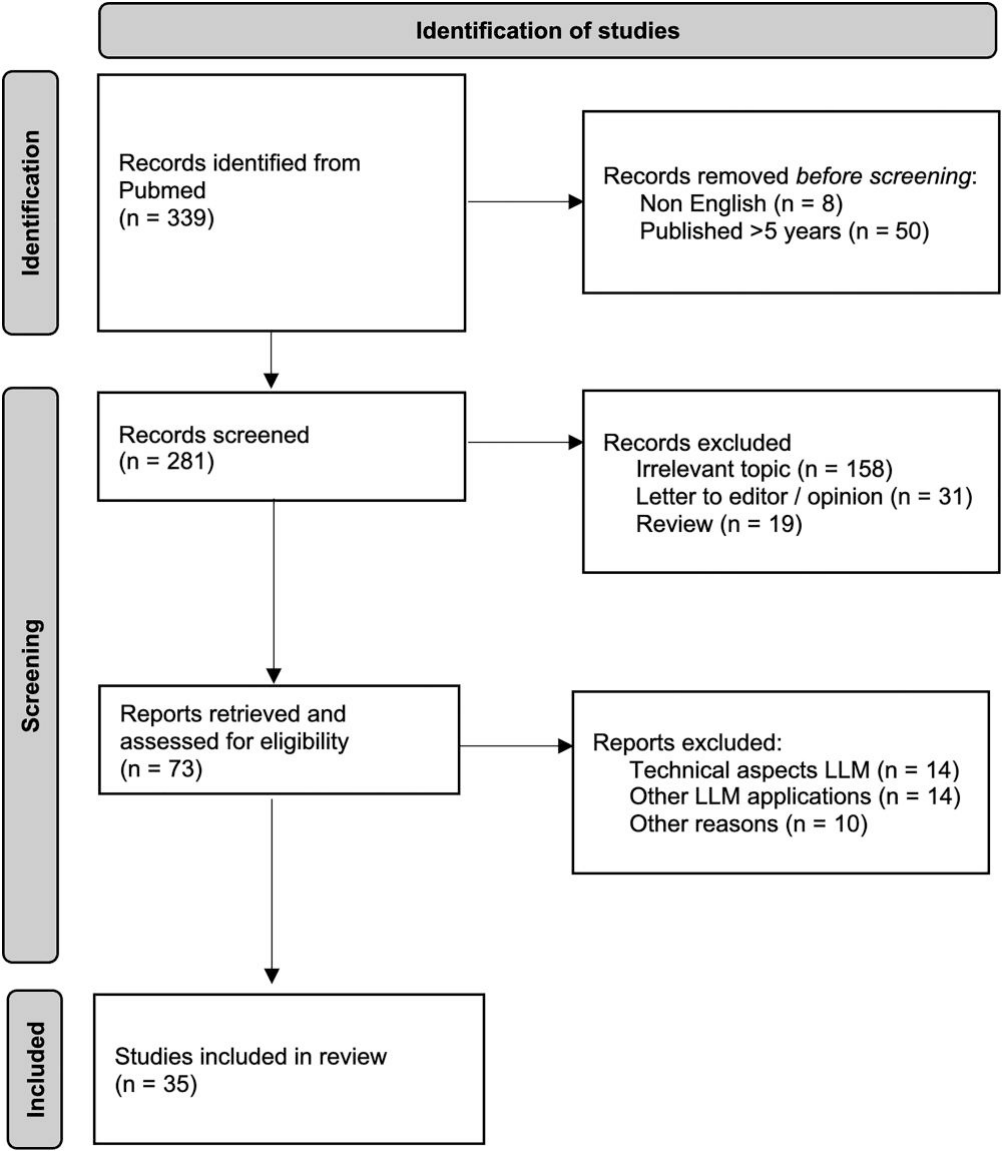
PRISMA flowchart for studies selection.

The study selection process is summarized in *Figure 1*. The initial PubMed search yielded 339 records, with 58 records excluded prior to screening for being in non-English languages (*n* = 8) or published more than 5 years ago (*n* = 50). A total of 281 studies were subsequently screened, of which 208 were deemed ineligible, leaving 73 studies for full-text review. Of these, 38 studies were further excluded: 14 focused on technical aspects of LLMs, 14 on natural language processing without using an LLM architecture, and 10 for mixed reasons, including the use of chatbots not powered by LLMs, LLMs applied to answering medical exams, and clinical trial design protocols. Ultimately, 35 studies met the inclusion criteria and were included in the systematic review.

### Study characteristics

The studies included in this review were published exclusively in 2023 and 2024, as no older studies met the inclusion criteria. They were conducted across multiple regions, with 43% from North America, 34% from Asia, and 14% from Europe (*Table 1*). Most were original research articles (*n* = 28, 80%), while a minority were shorter research letters (*n* = 7, 20%). All studies had an observational design, and the risk of bias was rated as high in most studies (*n* = 30), moderate in a few (*n* = 5), and low in only one study.

**Table 1:** General publication characteristics and risk of bias

Study ID	Author	Year	Country	Condition	LLM application	Risk of bias
**1**	Sarraju *et al*.^19^	2023	USA	CV prevention	Patient education	High
**2**	Kusunose *et al*.^20^	2023	Japan	Hypertension	Clinical support	High
**3**	O’Hagan *et al*.^21^	2023	Australia	Hypertension	Patient education	High
**4**	Huang *et al*.^22^	2023	China	Diabetes	Patient education	High
**5**	Fernández-Cisnal *et al*.^23^	2023	Spain	General cardiology	Patient education	High
**6**	Hulman *et al*.^24^	2023	Denmark	Diabetes	Patient education	Moderate
**7**	Yavuz and Kahraman^25^	2023	Turkey	General cardiology	Clinical support	High
**8**	Azizi *et al*.^26^	2024	Canada, USA	Atrial fibrillation	Patient education and clinical support	High
**9**	Birkun and Gautam^27^	2024	Russia, India	ACS	Patient education	High
**10**	Hong *et al*.^28^	2024	USA	Diabetes	Patient education	High
**11**	Yano *et al*.^29^	2024	Japan	Hypertension	Patient education	High
**12**	Barlas *et al*.^30^	2024	Turkey	Obesity	Patient education	High
**13**	Mondal *et al*.^31^	2024	India	CV prevention	Patient education	High
**14**	Hillmann *et al*.^32^	2024	Germany	Atrial fibrillation, devices^b^	Patient education	Moderate
**15**	Zaleski *et al*.^33^	2024	USA	Exercise	Patient education	High
16	Gurbuz *et al*.^34^	2024	Turkey	ACS	Patient education and clinical support	High
**17**	Motaghi Niko *et al*.^35^	2024	Iran	Hypertension	Patient education	High
**18**	Almagazzachi *et al*.^36^	2024	USA	Hypertension	Patient education	High
**19**	Dimitriadis *et al*.^37^	2024	Greece	Heart failure	Patient education	High
**20**	Dergaa *et al*.^38^	2024	Multiple^a^	Exercise	Patient education and clinical support	High
**21**	Al Tibi *et al*.^39^	2024	USA	Hypertension	Clinical support	High
**22**	Salihu *et al*.^40^	2024	Switzerland	Valvular disease	Clinical support	Moderate
**23**	Pham *et al*.^41^	2024	USA	ACLS	Clinical support	Moderate
**24**	Kozaily *et al*.^42^	2024	USA	Heart failure	Patient education	High
**25**	Lee *et al*.^43^	2024	USA	Hypertension	Patient education	High
**26**	Neo *et al*.^44^	2024	Singapore	Stroke rehabilitation	Patient education	High
**27**	Lee *et al*.^45^	2024	USA	Hyperlipidaemia	Patient education	High
**28**	King *et al*.^46^	2024	USA	Heart failure	Patient education	High
**29**	Lee *et al*.^47^	2024	USA	Atrial fibrillation	Patient education	High
**30**	Chung and Chang^48^	2024	Republic of Korea	Diabetes	Patient education	High
**31**	Vyas *et al*.^49^	2024	USA	Atrial fibrillation	Patient education	High
**32**	Li *et al.*^50c^	2024	China	Diabetes	Clinical support	Low
**33**	Naja *et al*.^51^	2024	Lebanon	Diabetes	Patient education	High
**34**	Anaya *et al*.^52^	2024	USA	Heart failure	Patient education	High
**35**	El Hajjar *et al*.^53^	2024	USA	Atrial fibrillation	Patient education and clinical support	High

CV, cardiovascular; ACS, acute coronary syndromes; ACLS, advanced cardiovascular life support.

^a^International collaboration (countries not specified).

^b^Cardiac implantable devices.

^c^All studies were observational in design (descriptive and cross-sectional), except this that employed a prospective cohort design.

### Type of large language models under evaluation

Most of the studies evaluated commercially available LLMs, specifically ChatGPT in 32 studies (91%), Bing Chat (powered by GPT-4) in 5 studies, and Gemini (or former Google Bard) in 5 studies (*Tables 2* and *3*; Supplementary material online, *Tables S2–S7*). Among the ChatGPT studies, 21 used version 3.5, 9 used version 4, and 2 studies compared both versions. In seven studies (20%), ChatGPT was compared with Bing Chat and/or Gemini (or Bard). Only one study evaluated a non-commercial LLM, the DeepDR-LM system, which was specifically developed for retinopathy screening using a machine learning algorithm combined with an LLM fine-tuned from Large Language Model Meta AI (LLaMA).^50^

**Table 2:** Summary of objectives, methods and key findings of publications evaluating large language models on cardiovascular risk factors

Study ID	Objective	LLM type and evaluation date^a^	Intervention	Key findings
**1**	Assess CVD prevention recommendations provided by ChatGPT	ChatGPT-3.5	25 questions related to basic CVD prevention	21 out of 25 responses (84%) were rated as appropriate
		December 2022		4 responses (16%) were inappropriate, primarily due to potential misinformation
**2**	Evaluate ChatGPT's responses to clinical questions on hypertension guidelines	ChatGPT-3.5	31 clinical questions from Japanese Society of Hypertension guidelines	Overall accuracy 64.5%
		April 2023		7 (out of 31) responses were inconsistent when repetitive prompting was used
**3**	Evaluate ChatGPT's responses to common hypertension-related patient questions	ChatGPT-3.5	15 FAQ on hypertension	Average readability above the recommended grade level; lack of clear credibility (JAMA criteria). Inaccuracies diminished with prompting over time
		February, April, and May 2023		
**4**	Evaluate ChatGPT's responses to common patient diabetes-related questions	ChatGPT-3.5	12 FAQ on diabetes	ChatGPT provided highly accurate responses for most questions; 3 questions scored a perfect 10 and the remaining 9 had an average score of 9.5 ± 0.2
		July 2023		
				Average reading grade was higher than recommended
**6**	Evaluate whether healthcare professionals can distinguish between answers about diabetes provided by ChatGPT vs. human experts	ChatGPT-3.5	10 FAQ on diabetes; ChatGPT answer compared with human expert by 183 professionals	Participants correctly identified ChatGPT answers 59.5% of the time (outside of the predefined non-inferiority margin of 55%
		January 2023		
**10**	Evaluate ChatGPT's responses to common patient diabetes-related questions	ChatGPT-3.5	25 FAQ on diabetes	19 responses (76%) were deemed appropriate by consensus
		March 2023		84% of the responses included a sentence stating the importance of discussing with a healthcare provider
**11**	Evaluate ChatGPT's responses to common hypertension-related patient questions posed in both Japanese and English	ChatGPT-4	20 FAQ on hypertension	85% of ChatGPT's answers were appropriate (Gwet's agreement coefficient 0.890, *P* < 0.0001)
		August 2023		
				Answers in English were more accurate and comprehensive and had more detail
**12**	Evaluate ChatGPT's responses for assessing obesity questions in diabetics	ChatGPT-3.5	20 questions on obesity	All responses in the general section were compatible with guidelines; four in six nutrition and physical activity responses were compatible with the guidelines, one was insufficient, and one was deemed incompatible; two out of five pharmacotherapy responses were accurate but incomplete
		April 2023		
**13**	Evaluate ChatGPT's responses to lifestyle-related diseases	ChatGPT-3.5	20 lifestyle-related disease/disorder case vignettes, each with four specific questions (including obesity, diabetes, and CVD)	Accuracy score was 1.83 ± 0.37 out of 2, with most responses considered accurate; there were no inaccurate responses
		July 2023		
**15**	Assess individualized exercise recommendations generated by ChatGPT for various clinical populations	ChatGPT-3.5	Individualized exercise recommendations for 26 clinical populations (including CDV, diabetes, hypertension, and other)	41.2% of exercise recommendations were comprehensive and 90.7% were accurate
		March 2023		
				Average reading grade was college-level and text classified as ‘difficult to read’; there answers with potential bias and discrimination
**17**	Compare ChatGPT and Bing in responding to Home Blood Pressure Monitoring knowledge	ChatGPT-3.5 and Bing	10 FAQ on Home Blood Pressure Monitoring Checklist	ChatGPT had a mean accuracy score of 5.96, while Bing achieved 5.31
		May 2024		ChatGPT outperformed Bing in accuracy, completeness, and consistency
**18**	Evaluate ChatGPT's responses to common hypertension-related patient questions	ChatGPT-3.5	100 FAQ on hypertension	93% of the questions had reproducible responses and overall ChatGPT had an accuracy of 92.5%
				Inappropriate responses were related to more complex or individualized clinical interpretation questions
**20**	Evaluate exercise prescriptions generated by GPT-4 for patients with diverse health conditions	ChatGPT-4	ChatGPT was tasked with creating a 30-day exercise programme using FITT principle (for five hypothetical patients, including hypertension and diabetes)	ChatGPT generated safe, conservative exercise programmes emphasizing moderate-intensity workouts
		June 2023		
				Programmes lacked precision in tailoring exercise to individual needs and tended to overemphasize safety
**21**	Compare medication recommendations between a cardiologist and ChatGPT-4 for hypertension patients	ChatGPT-4	40 hypertension patients; comparison of ChatGPT vs. cardiologist recommendations on medication	95% of patients had conflicting recommendations with ChatGPT-4 recommending significantly more medication changes (102 vs. 49 by the cardiologist)
				No agreement between ChatGPT-4 and the cardiologist (Cohen's kappa coefficient was −0.0127)
**25**	Compare ChatGPT vs. Google Gemini in responses to common hypertension-related patient questions	ChatGPT-3.5	52 FAQ on hypertension	ChatGPT was more likely to give a partially correct response (vs. Gemini, *P* = 0.035)
		Gemini-1.0		
		September 2023		Responses were shorter with ChatGPT but required a higher reading grade
**27**	Compare ChatGPT versions 3.5 and 4.0 when answering FAQ on hyperlipidaemia	ChatGPT-3.5	25 FAQ on hyperlipidaemia	ChatGPT-4.0 had a higher percentage of correct responses (74.67%) compared with ChatGPT-3.5 (69.33%)
		ChatGPT-4		Both versions provided reliable information, with incorrect responses being rare (5% or less); ChatGPT-4.0 offered more concise and readable responses
		May 2024		
**30**	Evaluate ChatGPT's responses to exercise-related questions for patients with type 2 diabetes	ChatGPT-4	14 FAQ on exercise for managing type 2 diabetes	71.4% of responses were rated as completely accurate and 28.6% were rated as accurate but incomplete
		November 2023		
				All responses scored 4/4 for safety and usefulness
**32**	Evaluate a DeepDR-LLM system that combines a large language model and deep learning model for diabetic retinopathy screening and diabetes management	DeepDR-LLM^b^	Two-arm prospective study; 785 patients with diabetes and gradable fundus images were evaluated by 12 PCP (unassisted vs. DeepDR-LLM assisted)	Patients evaluated by a DeepDR-LLM-assisted PCP had enhanced self-management behaviours in newly diagnosed diabetes (*P* < 0.05), earlier referral to an ophthalmologist if DR was present (4 days vs. 7 days, *P* < 0.001)
		April–July 2023		
				PCP reported higher satisfaction (score of 4.50 out of 5)
**33**	Evaluate ChatGPT responses in nutritional management for type 2 diabetes and MetS	ChatGPT-3.5-turbo	63 questions on nutrition management for diabetes and MetS patients	ChatGPT clarity was rated as good or excellent, but significant gaps in accuracy for critical nutrition advice were identified
		October 2023		
				ChatGPT menus deviated from the specified caloric intake and failed to meet several dietary recommendations

FAQ, frequently asked questions; vs., versus; PCP, primary care physician; MetS, Metabolic Syndrome; CVD, cardiovascular disease; FITT, frequency, intensity, time, type.

^a^If date was not reported by the authors, it was left in blank.

^b^Proprietary integrated image-language system; the LLM was fine-tuned from LLaMA.

**Table 3:** Summary of objectives, methods, and key findings of publications evaluating large language models on cardiovascular diseases

Study ID	Objective	LLM type and evaluation date^a^	Intervention	Key findings
**8**	Compare ChatGPT and Bing AI for patient and clinician inquiries on AF	ChatGPT-3.5	36 AF-related questions (18 patient questions tested on ChatGPT and 18 clinical questions tested on both models)	Appropriate responses in 83.3% of patient questions
				Correct responses in 33.3% for text accuracy and 55.5% for reference accuracy in ChatGPT clinical questions; Bing AI performed similarly (66.6% in response accuracy and 50% in reference accuracy)
		Bing Chat (GPT-4)		
**14**	Evaluate responses by different chat-based AI models (Google Bard, Bing Chat, and ChatGPT Plus) regarding AF and implantable cardiac devices	Google Bard	50 patient-centred questions (25 on AF and 25 on implantable cardiac devices)	For AF questions, appropriateness for ChatGPT was 84%, Bing Chat 60%, and Google Bard 52%
		Bing Chat (GPT-4)		
		ChatGPT-4.0		For implantable cardiac devices questions, appropriateness for ChatGPT was 88%, Bing Chat 72%, and Google Bard 16%
		May–October 2023		
				Google Bard showed the better readability
**29**	Evaluate ChatGPT responses on AF patient education	ChatGPT-3.5	16 FAQ on AF	85.9% of ChatGPT were correct and 4.7% perfect; 1.6% of responses were incorrect and 7.8% partially correct
			Four prompt formats (no prompt, patient-friendly prompt, physician-level prompt, and statistics and references prompting)	
				No difference in responses across different prompts (*P* = 0.350)
				ChatGPT provided references in only three (4.7%) responses
**31**	Evaluate ChatGPT's responses to common questions about AF	ChatGPT-3.5	20 AF-related questions	55.5% of answers were rated either ‘excellent’ or ‘very good’; 7.7% were rated poor (7.7%)
		November 2023		
**35**	Compare different LLMs in answering AF related questions	ChatGPT-4	20 patient-centred questions and 20 physician-centred questions	ChatGPT-4 2024 was the best model (patient questions were 90% accurate; physician questions were 55% accurate, 35% accurate but incomplete, and 10% inaccurate)
		Gemini-1.0		
		June 2023 and January 2024		
				Gemini showed lower overall accuracy than ChatGPT-4 (33 vs. 73%, *P* < 0.01); ChatGPT-4 accuracy improved over time (45% in 2023 vs. 73% in 2024)
**19**	Evaluate ChatGPT's responses to FAQ on HF	ChatGPT-3.5	47 FAQ on HF	ChatGPT provided correct and comprehensive answers for 41 out of 47 questions (87%)
				Responses were consistent across repeated prompts
**24**	Evaluate the potential of LLM-based AI chat platforms in answering patients questions on HF	ChatGPT-3.5	30 FAQ on HF	ChatGPT-3.5 provided 90% accurate answers (27/30), while Bard provided 56% (17/30)
		Google Bard		
		June 2023		Bard occasionally hallucinated references or underplayed risks
**28**	Evaluate ChatGPT's responses to FAQ on HF	ChatGPT-3.5	107 FAQ on HF	GPT-4 outperformed GPT-3.5, providing 100% correct responses and 83.2% graded as comprehensive (98.1% and 78.5%, in GPT-3.5, respectively)
		ChatGPT-4		
				Both models demonstrated high reproducibility
				GPT-4 did not give any incorrect information
**34**	Evaluate ChatGPT's responses to FAQ on HF	ChatGPT-3.5	12 FAQ on HF	ChatGPT's responses were longer and more challenging to read, compared with AHA/ACC/HFSA educational materials
		November 2023		
				ChatGPT's output included a high percentage of difficult words; actionability score was 67%
**5**	Evaluate Bing Chat performance in providing assistance for common cardiovascular conditions	Bing Chat (GPT-4)	14 simulated patients with cardiovascular-related health conditions using a freestyle-like conversation	Bing provided appropriate and safe final advice in all 14 cases (100%)
				An appropriate anamnesis was found in 10 out of 14 cases (71%)
		February 2023		93% of the responses were rated as clear and easy to understand
**7**	Evaluate ChatGPT-4.0 performance in providing pre-diagnosis and treatment plans for cardiac clinical cases	ChatGPT-4	20 cardiology clinical cases (developed by experienced cardiologists)	ChatGPT-4.0 had a high physician adherence rate to diagnoses (median 5.00, IQR 1)
				ChatGPT-4.0's management plan received a median score of 4 (IQR 1), indicating a good quality of response as perceived by the physicians
**9**	Evaluate the quality of first aid advice provided by Bing Chat for heart attack queries in three countries	Bing Chat (GPT-4)	A ‘heart attack what to do’ query (simulating users seeking first aid advice) was done in three countries (Gambia, India, and USA)	Full congruence (completely satisfied) with checklist items was low, ranging from 7.3% in India, 8.6% in Gambia, and 16.8% in the USA
		May 2023		
				The readability differed, being lower for the Gambia and the USA than for India (*P* = 0.008); omissions and inaccuracies were frequent
**16**	Evaluate ChatGPT's responses to FAQ on ACS	ChatGPT-3.5	72 FAQ on ACS (patient and clinical questions)	ChatGPT achieved high accuracy, with 65 (90.3%) of its responses scoring GQS 5 (highest accuracy and proficiency). None of the responses scored GQS 1 (lowest); reproducibility was high (94.4%)
**22**	Ability of ChatGPT to support clinical decision in severe AS	ChatGPT-4	ChatGPT was asked treatment recommendations based on 150 patient with severe AS discussed in heart team meetings	ChatGPT's decisions agreed with Heart Team decisions 77% of cases
				Agreement rate was 90% for TAVI, 65% for SAVR, and 65% for medical treatment; 35 patients were misclassified
**23**	Assess accuracy of ChatGPT to advanced cardiovascular life support guidelines	ChatGPT-4	2 simulated clinical scenarios (cardiac arrest and bradycardia management)	Median overall accuracy for cardiac arrest was 69% (IQR 67–74%) and for bradycardia 42% (IQR 33–50%)
		May–August 2023		
				A lack of step-by-step guided consistency was found
**26**	Evaluate two LLMs in responding to rehabilitation concerns from stroke survivor patients and caregivers	ChatGPT	10 questions curated from stroke patients and caregivers	ChatGPT received 79 satisfactory grades (65.8%) and Bard received 91 (75.8%)
		Google Bard		Both chatbots demonstrated good readability (90% in ChatGPT and 86.7% in Google Bard)
		February 2024		
				Both chatbots had hallucinations and performed poorly in recognizing emotional or mental health risks

AF, atrial fibrillation; FAQ, frequently asked questions; AI, artificial intelligence; AHA/ACC/HFSA, American Heart Association/American College of Cardiology/Heart Failure Society of America; ACS, acute coronary syndromes; ESC, European Society of Cardiology; AS, aortic stenosis; TAVI, transcatheter aortic valve implantation; SAVR, surgical aortic valve replacement; AHA, American Heart Association; ACLS, advanced cardiovascular life support.

^a^If date was not reported by the authors, it was left in blank.

### Applications of large language models in cardiovascular disease and risk factors

**Figure 2:**
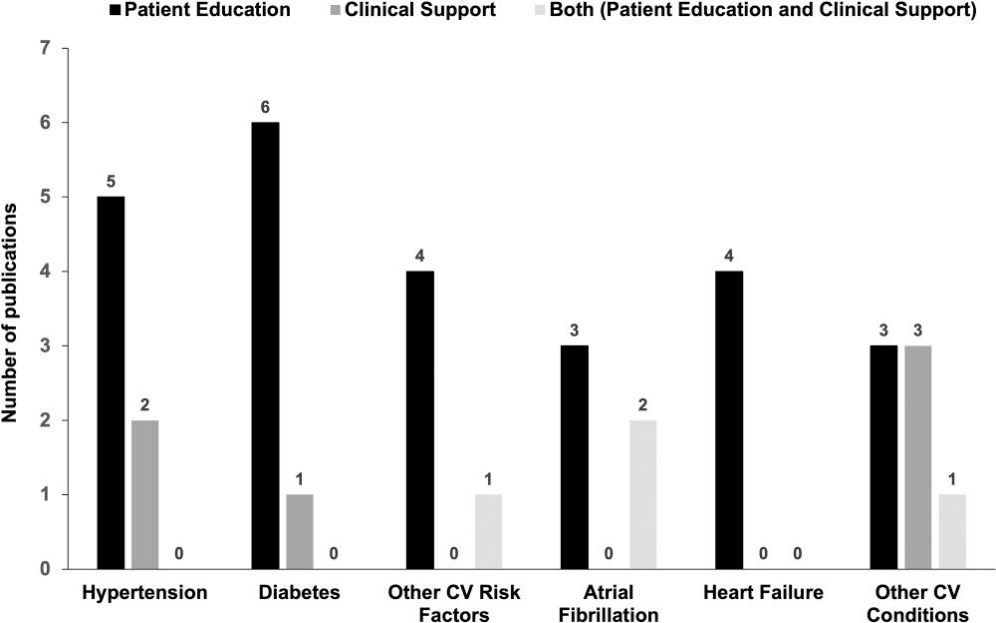
Distribution of publications according to the conditions evaluated in large language models’ application.

Most studies evaluated LLMs in the context of primary CVD prevention and risk factors (54%), with hypertension and diabetes being the most frequently addressed (*Table 1* and *Figure 2*; Supplementary material online, *Tables S2–S4*). The remaining studies (46%) focused on other CVDs, including atrial fibrillation and heart failure (*Table 2* and *Figure 2*; Supplementary material online, *Tables S5–S7*).

**Figure 3:**
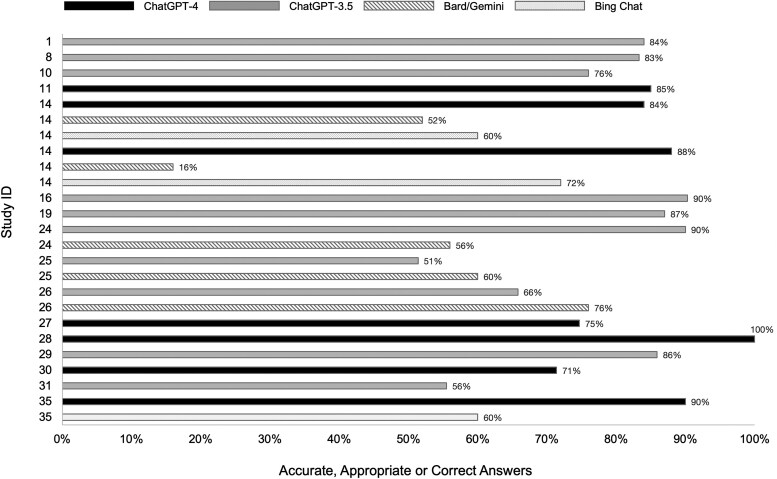
Large language models’ performance in answering the most frequent questions asked by the patients*. *Only studies reporting accurate, appropriate, or correct answers are included; study ID 14 includes two separate entries, one for atrial fibrillation–related questions and another for implantable cardiac devices.

The applications of LLMs were broadly categorized into two main areas: patient education (*n* = 25, 72%) and clinical decision support (*n* = 6, 17%), with a smaller proportion (*n* = 4, 11%) addressing both areas (*Table 1* and *Figure 2*). The primary application of LLMs was answering frequently asked patient questions, evaluated in 24 studies (68%).^19,21,22,24,26,28–32,34–37,42–49,52,53^ All these studies used ChatGPT (version 3.5 or 4), with seven of these comparing it with other commercially available models (Gemini or Google Bard and/or Bing Chat). Overall, the studies concluded that LLMs generally provided accurate, appropriate, or correct answers, although performance varied by model (*Figure 3*). The lowest-performing LLM in terms of providing appropriate answers was Google Bard, as reported in a study by Hillman *et al*.,^32^ which found its accuracy to be 52% for atrial fibrillation–related questions and only 16% for questions concerning implantable cardiac devices. When compared with other LLMs, ChatGPT generally outperformed them in appropriateness, accuracy, and completeness when responding to patient questions.^32,35,42–44,53^ Additionally, two studies examined ChatGPT’s performance over time, showing an improvement in the correctness and accuracy of answers, especially when comparing version 3.5 with version 4.^21,46^

Large language models generally demonstrated consistency when prompted multiple times and with varying prompt structures.^19,35–37,46,47^ Although most responses were correct, a variable proportion of responses contained incomplete content, lacking critical information, particularly regarding new guideline-based management strategies (e.g. omitting SGLT-2 inhibitors in heart failure with preserved ejection fraction), newer drugs (e.g. missing semaglutide and inclisiran), and specific procedures (e.g. ChatGPT's had difficulty in distinguishing between medical devices and metabolic surgery).^19,22,30,32,36,37,42^ Additionally, some answers that required more context or individualized interpretation also lacked information, as seen in questions like ‘What is the normal blood pressure range for a 65-year-old?’ or ‘What blood pressure range should I maintain with chronic kidney disease?’.^36^

Some answers contained misinformation (e.g. LLM responded to questions about exercise by firmly recommending both CV activity and lifting weights, which may not always be appropriate), but completely incorrect responses were rare.^19,22,26,30^ For instance, in response to ‘Can diabetes be ruled out if fasting blood sugar is normal?’, ChatGPT incorrectly cited a range of 70–100 mg/dL, and for nutritional advice for obese diabetic patients, it recommended unsupported supplements.^22,30^ Hallucinations were uncommon and mainly involved erroneous references.^26,42,44^ When explicitly prompted for sources, references were provided in only a few responses.^21,47^ In questions involving clinical decisions, LLMs frequently included a recommendation to consult a healthcare provider.^28,32^

Large language model–generated text typically required a high school to college-level comprehension grade and was classified as difficult to read, with a higher word count compared with patient materials provided by scientific societies.^21,31,32,45,52,53^

In one study evaluating ChatGPT-4’s responses to 600 patient questions on atrial fibrillation, 30 experienced physicians rated only 7.7% of the answers as ‘poor’. Additionally, 67% of the participating physicians considered ChatGPT a reliable source of information for patients, and 60% stated that its responses were comparable with those provided by practicing clinicians.^49^ Interestingly, in a study where 183 healthcare professionals from a diabetes centre were asked to classify answers to patient questions as either human or ChatGPT-generated, participants correctly identified ChatGPT answers 60% of the time. Although this exceeds the 50% accuracy expected by chance, it fell short of the predefined non-inferiority margin of 55%.^24^

Beyond using LLMs to answer frequently asked patient questions, some studies have explored other applications. In one study, Bing Chat was utilized to assist with 14 common CV conditions (including syncope, paroxysmal tachycardia, aortic stenosis, heart failure, and chest pain) through a freestyle conversational approach.^23^ Two experienced cardiologists reviewed the responses, concluding that all cases (100%) provided appropriate and safe final advice. Additionally, the chatbot was rated as offering an appropriate anamnesis in 10 out of 14 cases (71%), and 93% of responses were deemed clear and easy to understand.^53^

One study evaluated Bing Chat’s ability to advise patients on first aid for heart attack symptoms by testing the query ‘heart attack what to do’ in three different countries.^27^ The responses were inconsistent and showed low compliance with guidelines, with adherence rates of 7.3% in India, 8.6% in Gambia, and 16.8% in the USA. Common omissions included critical life-saving actions, such as initiating cardiopulmonary resuscitation for an unresponsive person or calling emergency medical services. Inaccuracies were also noted, such as referencing incorrect emergency numbers and advising users to open windows.

In two studies, ChatGPT was prompted to provide individualized exercise prescriptions for various patient populations, including those with CVD such as hypertension and diabetes.^33,38^ While the exercise plans were generally accurate, they lacked comprehensive customization for the individual. Large language models’ responses often overemphasized safety, limiting training progression and intensity, and recommended medical clearance even for low-risk individuals. In the study by Dergaa *et al*.,^38^ the FITT principle (Frequency, Intensity, Time, Type) was used, and results suggest that ChatGPT could be a useful tool for physicians seeking guidance on exercise prescription. In one study, ChatGPT was utilized for dietary management, providing nutrition recommendations and menu planning for patients with diabetes and metabolic syndrome.^51^ While the responses were rated as good or excellent overall, significant gaps in accuracy were noted, including deviations from the specified caloric intake and failure to meet several key dietary recommendations.

In the clinical decision support arena, four studies evaluated LLMs’ performance in assisting with medical questions.^20,26,34,53^ In two studies involving atrial fibrillation–related questions tested on ChatGPT, Bing AI, and Gemini, accuracy rates were lower compared with patient-focused questions, with ChatGPT-3.5 and Gemini achieving 33%, Bing Chat 67%, and ChatGPT-4 73%.^26,53^ Reference accuracy averaged 50%, with occasional fabricated references.^26^ In a study evaluating ChatGPT’s responses to acute coronary syndrome questions based on European Society of Cardiology guidelines, 88% of answers achieved the highest accuracy and proficiency score.^34^ ChatGPT also scored 65.5% accuracy on hypertension guideline questions, with lower accuracy (36%) in areas lacking strong evidence-based guidelines.^20^

Commercially available LLMs have also been tested for their ability to assist in medical diagnosis and management planning. ChatGPT-4 demonstrated high diagnostic performance and good quality in treatment suggestions across 20 cardiology cases, performing well regardless of case complexity.^25^ In a study comparing ChatGPT-4’s treatment strategy decisions with heart team recommendations for 150 patients with valvular heart disease, concordance was 77%, including 90% agreement for transcatheter aortic valve implantation, 65% for surgical aortic valve replacement, and 65% for medical management.^40^ Notably, ChatGPT-4 outperformed a guideline-based decision tree, showing higher concordance and accuracy. Conversely, other studies reported lower performance and accuracy of this model when used for clinical decisions. For instance, treatment recommendations by ChatGPT-4 for 40 hypertension patients conflicted with a cardiologist’s decisions in 95% of cases.^39^ ChatGPT-4 was also evaluated for step-by-step guidance in advanced cardiac life support protocols, with median accuracy scores of 69% (IQR 67–74%) for cardiac arrest and 42% (IQR 33–50%) for bradycardia.^41^ Critical actions, such as establishing intravenous access and obtaining an electrocardiogram, were often omitted, with repetitive emphasis on some actions (e.g. ‘check rhythm’) and errors in medication dosages (e.g. incorrect atropine doses). The model could not provide a consistent step-by-step guide for managing these scenarios.

Finally, in one study evaluating a non-commercial LLM, the DeepDR-LM system, 785 diabetic patients were assessed for adherence to recommendations and retinopathy screening followed by tertiary referral.^50^ Physicians using this proprietary LLM reported high satisfaction, noting it to be understandable, time-saving, effective, and safe in clinical practice. Newly diagnosed diabetes patients demonstrated improved self-management behaviours at 4 weeks (*P* < 0.05), and among those with retinopathy, there was a more timely referral to an ophthalmologist (*P* < 0.001) when physicians were assisted by DeepDR-LM.

## Discussion

This systematic review offers a comprehensive assessment of LLMs’ applications in CVD, including risk factor management. Commercially available LLMs, particularly ChatGPT, demonstrate significant potential in patient education by providing reliable, consistent, and generally safe responses to common queries. However, substantial variability exists in the accuracy, appropriateness, and depth of responses among different LLM platforms, with performance influenced by prompting techniques. Beyond patient education, LLMs hold promise in supporting clinical decision-making. Nonetheless, there is a critical need for specialized LLMs designed to integrate patient data and provide personalized, state-of-the-art recommendations.

To our knowledge, this is the first systematic review to explore a broad range of LLM applications in the prevention and treatment of CVD. Although there is considerable enthusiasm and promising advancements regarding the use of LLMs in CV medicine, with recent review papers showcasing titles such as ‘Artificial Intelligence: Revolutionizing Cardiology with Large Language Models’ and ‘Maximizing Large Language Model Utility in Cardiovascular Care’, our findings indicate that substantial work remains to be done before LLMs can be incorporated in routine clinical practice.^2,6^ Notably, our PubMed search for LLM-related keywords yielded only 9825 results, compared with 2 156 715 results for CVD terms, with only 339 articles addressing both topics combined (see Supplementary material online, *Table S1*). The current literature on LLMs’ applications in CVD primarily consists of observational and exploratory studies, lacking the scientific rigour required to change clinical practice.

Our review found limited evidence for using LLMs in clinical decision support, with only 10 studies (29%) reporting applications in this area. Some studies suggest that ChatGPT and similar LLMs can successfully pass various medical examinations, demonstrating extensive knowledge, even with general-purpose LLMs not specifically developed for medical applications.^13^ However, this application of LLMs was not included in our search strategy, as it was assumed to lack direct implications for clinical practice and patient education. Nevertheless, we identified four studies indicating that commercially available LLMs, including ChatGPT, Bing Chat, and Gemini, can aid clinicians in addressing clinical questions, with some limitations.^20,26,34,53^ Also of interest, LLMs show potential utility, supporting doctors in exercise prescription and nutrition plan preparation; while these responses lack full personalization, they provide useful templates that can be refined and validated by the practicing physician.^38,51^ Another promising area for LLM support is the analysis of individual patient data to formulate treatment recommendations. Notably, one study evaluated ChatGPT-4 performance in heart team decision-making for patients with severe aortic disease. The results were noteworthy, with ChatGPT achieving a 77% concordance with clinician decisions and outperforming strict guideline-based decision tree criteria.^40^ Discordance was most evident in complex cases, particularly those involving decisions between medical management and surgical aortic valve repair, areas where heart teams themselves often face challenges in determining the best approach.

Remarkably, a proof-of-concept study included in our review demonstrates that a non-commercial LLM specifically designed for diabetic patients can enhance physician efficiency and reduce consultation time without compromising patient safety.^50^ While there is still room for improvement, refining commercially available LLMs or developing medical-specific models could provide valuable support for physicians in daily practice. Nonetheless, critical thinking remains an essential skill, especially given the inaccuracies present in these models, which can directly impact patient care.^2,3,6^

An important area for LLM applications is health literacy and patient education, both essential components of effective clinical care. Patients frequently seek information about their medical conditions online, turning to search engines (e.g. Google, Yahoo, and Microsoft Bing), health-focused websites like WebMD, and medical society websites that prioritize accurate and up-to-date content. A systematic review by Sharma *et al*.,^54^ conducted between November 2022 and September 2023, examined ChatGPT applications in cardiology and found that only 3 out of 24 publications addressed its use in patient education. In contrast, our review identified 29 studies focusing on patient education, with 24 evaluating LLMs’ ability to answer frequently asked patient questions, highlighting the growing interest in this field. Our findings suggest that commercially available LLMs could serve as an alternative source for patient information. Several studies report high accuracy (over 90% complete and correct answers) and comprehensive, reproducible responses to common patient queries, free from incorrect or unsafe information.^34,42,46,53^ Furthermore, in a study evaluating physicians’ perspectives on LLM responses for atrial fibrillation, over 60% of clinicians considered these responses reliable and comparable with those provided by healthcare professionals.^49^ In another study on diabetes-related frequently asked questions, 40% of healthcare professionals, when blinded to the response source, could not differentiate between human-generated and ChatGPT-generated responses.^24^ Hallucinations, a primary concern with LLMs, were found to be uncommon and mainly involved erroneous references, a recognized limitation of commercially available models.^26,42,44^ Establishing robust mechanisms to validate sources in LLM outputs is essential, especially in healthcare, where misinformation could compromise clinical decision-making and patient safety.^2,6^

While general-purpose, commercially available LLMs can serve as a growing source of information for patients, our systematic review highlights several limitations that must be acknowledged. Large language models are trained on data sets that may not stay current in rapidly evolving fields such as CV medicine, potentially leading to outdated or incomplete information over time (e.g. missing information on SGLT-2 for treating heart failure; semaglutide use in obesity).^30,37^ Furthermore, answers provided by LLMs were reviewed by a limited number of experts (only four studies in our review employed more than three reviewers), relying on subjective criteria with possible evaluator bias and lacking any patient feedback.^22,24,28,49^ Additionally, the readability of LLM-generated responses often exceeded the recommended 6th–8th-grade level for patient education, which may limit accessibility for individuals with lower health literacy. Interestingly, the mean readability grade level of patient education materials in high-impact journals also exceeds recommendations, ranging from grades 11.2 to 13.8, slightly below the levels observed in LLM responses in our review.^21,31,45,47,55^ The absence of visual or interactive content in text-based LLM responses may further impact patient comprehension.

Another limitation in validating LLMs for patient education is that they were tested using simulated questions, relying on a restricted set of common queries often designed by researchers, which may not fully capture the diversity of real-world patient inquiries. Although some studies incorporated prompt variation and rephrasing, most used single, precise, well-phrased questions, implicitly assuming that patients will not make errors or deviate from relevant content.^43,45,47^ Only one study employed a freestyle conversation approach, but this simulation was conducted by a physician rather than a patient.^23^ Large language models were primarily evaluated on common CV conditions, such as hypertension, diabetes, heart failure, and atrial fibrillation, whereas their performance in addressing questions related to more complex or rare conditions has yet to be assessed.

The application of LLMs in healthcare is constrained by limitations in language and regional applicability, as well as readability and accessibility challenges. Most studies have been conducted in English, which restricts the generalizability of findings to non-English-speaking populations and fails to consider regional variations in medical terminology, healthcare systems, and clinical guidelines, thereby reducing relevance across diverse geographic areas. For example, in the study by Birkun and Gautam,^27^ ChatGPT recommended that users in Gambia and India call 911, the US emergency number. Additionally, Yano *et al*.^29^ compared ChatGPT responses in English and Japanese for hypertension-related questions, and while accuracy was high in both languages, only 10% of the responses were rated as equally suitable in both Japanese and English, with the remaining 90% being more suited to English.

While the use of LLMs in patient literacy has been shown to provide accurate and comprehensive answers, there remains a risk of misinformation, and a lack of transparency regarding the sources used, with occasional hallucinations when models are asked to provide specific references.^19,21,22,26,30,42,44,47^ This issue could be mitigated by refining available models or developing specifically designed LLMs for the medical domain, such as Med-PaLM, although further investigation is warranted.^56^

This systematic review has several limitations that should be considered. First, our search was conducted exclusively in PubMed, and expanding to other databases may have yielded additional relevant articles. While we targeted studies published within the past 5 years, only studies from 2023 and 2024 met the inclusion criteria, underscoring the novelty of research on LLMs in CVD care and highlighting the potentially premature nature of this review. The included studies were predominantly observational, lacking experimental design, which limited our ability to conduct a meta-analysis and translate the findings to real-world practice. Moreover, the study design was not always clearly defined, making bias assessment challenging and impacting the robustness of our observations.

Additionally, our search terms did not include keywords for specific medical LLMs like Med-PaLM. Of notice, a PubMed search combining CVD terms with specialized medical LLMs returned no studies as of 10 November 2024 (see Supplementary material online, *Table S8*). Our systematic review focused on CV terms and may have overlooked other validated applications of LLMs. However, a recent review on the testing and evaluation of healthcare applications of LLMs did not identify significant studies within the CV field.^57^ Given the rapid evolution in artificial intelligence and LLM technology, new applications and research may have emerged since this review was completed, possibly broadening the scope of findings. A simple search on ClinicalTrials.Gov performed on 10 November 2024 returned 6381 registered trials using LLMs (see Supplementary material online, *Figure S1*).

## Conclusions

This systematic review underscores that while LLMs hold significant potential in CVD prevention and treatment, further rigorous testing and scientific validation are mandatory next steps. Evidence supporting the use of LLMs in patient education is growing, particularly as an alternative source of information for patients with common CVDs. Commercially available LLMs may serve as viable alternatives to traditional web searches, offering accessible answers to patients’ most frequently asked questions. However, employing LLMs to address individual patient needs, support diagnoses, and make treatment recommendations requires additional research. As LLM capabilities continue to evolve and specialized medical models are developed, a more comprehensive application of these tools in clinical and patient settings is anticipated.

## Supplementary Material

ztaf028_Supplementary_Data

## Data Availability

The data underlying this article are available in the article and in its online Supplementary material.

## Lead author biography

José Ferreira Santos is a consultant cardiologist and the chief medical officer at Hospital da Luz Setúbal in Portugal. With over 20 years of experience in clinical cardiology and healthcare leadership, Dr Santos specializes in cardiovascular imaging and preventive cardiology. He has authored 29 peer-reviewed papers and presented over 200 abstracts at international conferences and is currently a PhD student. He has a growing interest in innovative digital solutions to move forward cardiovascular prevention and is the founder of Cardio da Vida, a platform focused on cardiovascular disease prevention and public health education.
