# Supplementary material for: Applications of large language models in cardiovascular disease: a systematic review
Source: Eur Heart J Digit Health. 2025 Apr 1;6(4):540–53. doi: 10.1093/ehjdh/ztaf028 (PMC12282349; doi:10.1093/ehjdh/ztaf028)

**SUPPLEMENTARY MATERIAL**

**Table S1: Keywords used in article search strategy**

A comprehensive and automated search strategy was performed in Pubmed, using terms related to Large Language Models and Cardiovascular Disease, on October 20, 2024, using the following keywords.

| **#** | **Search Keywords (In Title)** | **PubMed**  **Results** |
| --- | --- | --- |
| 1 | "ChatGPT" OR "GPT" OR "GPT-3" OR "GPT-3.5" OR "GPT-4" OR "LLaMA" OR "LaMDA" OR "Large Language Model*" OR "Transformer Model*" OR "AI Chatbot*" OR "Natural Language Processing" OR “Chatbot*” OR “Language Model*” OR “Chat-Based” | 9,825 |
| 2 | “Cardiology” OR "Cardiovascular Disease" OR "Heart Disease*" OR “Heart” OR "Cardiac Disease" OR "Coronary Artery Disease" OR “Angina” OR “Acute Coronary Syndrome*” OR "Myocardial Infarction" OR "Heart Failure" OR "Atrial Fibrillation" OR "Stroke" OR "Peripheral Artery Disease" OR "Cardiomyopathy" OR "Valvular Heart Disease" OR “Heart Valve Disease*” OR "Congenital Heart Disease" OR "Risk Factors" OR "Hypertension" OR "Blood Pressure" OR "Diabetes" OR "Type 1 Diabetes" OR "Type 2 Diabetes" OR "Hyperlipidemia*" OR "Cholesterol” OR “Dyslipidemia" OR "Elevated Lipids" OR "Hypercholesterolemia" OR "Obesity" OR "Overweight" OR "Excess Weight" OR "Smoking" OR "Tobacco Use" OR "Cigarette Smoking" OR "Nicotine" OR "Lifestyle Interventions" OR "Diet" OR "Physical Activity" OR "Physical Inactivity" OR "Exercise" OR "Smoking Cessation" OR "Alcohol" OR "Weight" OR "Behav* Change" OR “Behavior Therapy” OR “Lifestyle” | 2,156,715 |
| 3 | 1 AND 2 | 339 |
| 4 | 1 AND 2 AND “English only” | 331 |
| 5 | 1 AND 2 AND “English only” AND “Last 5 years only” | 285 |

**Table S2.** Summary of objectives, methods and key findings of publications evaluating LLMs on hypertension

| Study ID | Objective | LLM Type & Evaluation Date^a^ | Intervention | Outcome Measures | Key findings |
| --- | --- | --- | --- | --- | --- |
| 2 | Evaluate ChatGPT’s responses to clinical questions on hypertension guidelines | ChatGPT-3.5  April, 2023 | - 31 questions (21 clinical questions and 10 clinical questions with limited evidence) from Japanese Society of Hypertension (JSH) 2019 guidelines - 1 reviewer compared ChatGPT answers with guidelines | - Accuracy of responses - Variability of answers (Shannon Entropy) | - Overall ChatGPT accuracy 64,5% - Higher accuracy for clinical questions (80%) compared with questions with limited evidence (36%), p = 0.005 - No significant difference in accuracy between responses in Japanese and English (65% vs. 58%, p = 0.602) - Of the 21 clinical questions, 9 had zero entropy, indicating consistent answers while 7 exhibited high entropy (>0.5), reflecting inconsistent responses with repetitive prompting |
| 3 | Evaluate ChatGPT’s responses to common hypertension-related patient questions | ChatGPT-3.5  February, April, and May 2023 | - 15 FAQ on Hypertension - Prompting in 3 different time periods - 2 reviewers compared ChatGPT answers with US and European guidelines | - Readability (Simple Measure of Gobbledygook (SMOG) Index) - Credibility (JAMA benchmark criteria, focusing on authorship, attribution, ownership, and currency). - Accuracy (compared with guidelines) | - Average readability was above the recommended grade level, with scores increasing over time: 13.5 in February, 14.2 in April, and 14.3 in May - No response fully met JAMA criteria for credibility; there was a lack of clear authorship and currency, limiting the overall credibility - In February, 5 out of 15 responses contained inaccuracies, 3 out of 15 in April and 1 out of 15 in May |
| 11 | Evaluate ChatGPT’s responses to common hypertension-related patient questions posed in both Japanese and English | ChatGPT-4  August, 2023 | - 20 FAQ on Hypertension - 3 reviewers evaluated the answers against expert opinion; all were unaware that ChatGPT-4 was used | - Accuracy (“appropriate” or “inappropriate”) - Japanese and English responses were compared | - 85% of ChatGPT’s answers were deemed appropriate by all three reviewers (Gwet’s agreement coefficient 0.890, p<0.0001) - 18 out of 20 questions were more accurate, comprehensive and with more detail in English; the remaining 2 questions were comparable in both languages |
| 17 | Compare ChatGPT and Bing in responding to Home Blood Pressure Monitoring knowledge | ChatGPT-3.5  Bing  May, 2024 | - 10 FAQ on Home Blood Pressure Monitoring Checklist - 3 reviewers evaluated the answers against expert opinion | - Comparison of ChatGTP Vs Bing - Accuracy (six-point Likert scale) - Completeness (three-point scale) - Reproducibility (similarity between repeated answers) | - ChatGPT had a mean accuracy score of 5.96, while Bing achieved 5.31; ChatGPT provided 58 out of 60 responses with the top accuracy score - ChatGPT achieved a mean completeness score of 2.93, with 93% of responses rated as fully complete, while Bing had a mean score of 2.13, with many responses missing essential details - ChatGPT provided consistent responses for 28 out of 30 repeated questions, while Bing had inconsistencies in 5 of the 30 repeated answers - ChatGPT outperformed Bing in both accuracy and completeness |
| 18 | Evaluate ChatGPT’s responses to common hypertension-related patient questions | ChatGPT-3.5 | - 100 FAQ on hypertension - Answers compared against American Heart Association and National Institutes of Health Guidelines | - Reproducibility (if the answers across three separate runs conveyed the same message) - Accuracy (appropriate or inappropriate) - Appropriateness of responses from a clinical standpoint | - 93% of the questions had reproducible, while 7% of the questions had irreproducible responses - Overall ChatGPT had an accuracy of 92.5% - Inappropriate responses were primarily related to questions requiring more context or individualized clinical interpretation^b^ |
| 21 | Compare medication recommendations between a cardiologist and ChatGPT-4 for hypertension patients | ChatGPT-4 | - 40 hypertension patients from a rural clinic, who had at least two serum lab review appointments and a cardiologist’s medication recommendation - ChatGPT-4 was tasked with making medication recommendations based on patient data | - Comparison of medication recommendations by both the cardiologist and ChatGPT-4 | - 95% of patients had conflicting recommendations with ChatGPT-4 recommending significantly more medication changes (102 Vs 49 by the cardiologist) - Cohen’s kappa coefficient was -0.0127 (no agreement between ChatGPT-4 and the cardiologist) - ChatGPT-4 recommended stopping medications in 16 cases, compared to 0 from the cardiologist |
| 25 | Compare ChatGPT versus Google Gemini in responses to common hypertension-related patient questions | ChatGPT-3.5  Gemini-1.0  September, 2023 | - 52 FAQ on Hypertension - Answers compared against the American College of Cardiology FAQ responses | - Accuracy (correct, partially correct, or incorrect) - Readability (Flesch-Kincaid grade level and word count) | - ChatGPT was more likely to give a partially correct response (Vs Gemini, p=0.035) - Gemini had 59.6% correct, 33.7% partially correct, and 6.7% incorrect responses - ChatGPT had 51.4% correct, 44.2% partially correct, and 4.3% incorrect responses - ChatGPT's mean FK grade level was higher than Gemini’s (15.92 vs. 13.50, p<0.0001) - Google Gemini had a higher average word count compared to ChatGPT (44.43 vs. 21.08 words, p<0.0001) |

^a^ If date not reported by authors was left in blank

^b^ Examples of questions with inappropriate responses: "What is the normal range of BP for a 65 and older?"; "What range of BP should I maintain if I was diagnosed with chronic kidney disease?"

FAQ, frequently asked questions; Vs, versus

**Table S3.** Summary of objectives, methods and key findings of publications evaluating LLMs on diabetes

| Study ID | Objective | LLM Type & Evaluation Date | Intervention | Outcome Measures | Key findings |
| --- | --- | --- | --- | --- | --- |
| 4 | Evaluate ChatGPT's responses to common patient diabetes-related questions | ChatGPT-3.5  July, 2023 | - 12 FAQ on diabetes - 5 reviewers evaluated the answers against expert opinion | - Accuracy (10 indicated high accuracy and scores below 6 indicated inaccuracies) - Consistency on repeated prompts (5 runs) - Length (number of sentences) and readability (Flesch-Kincaid Grade Level) | - ChatGPT provided highly accurate responses for most questions; 3 questions scored a perfect 10 and the remaining 9, had an average score of 9.5 ± 0.2 - Each response had approximately 157±29 words, and the Flesch-Kincaid Grade Level average 13.8±1.1 - Certain responses lacked completeness and had inaccuracies ^b^ |
| 6 | Evaluate whether healthcare professionals can distinguish between answers about diabetes provided by ChatGPT Vs human experts | ChatGPT-3.5  January, 2023 | - 10 FAQ on Diabetes, with two possible answers (one from a human expert and other from ChatGPT) - 183 healthcare professionals working on a diabetes center classified the answers | - Logistic regression to analyze whether professionals could correctly identify the ChatGPT-generated answers | - Participants correctly identified ChatGPT answers 59.5% of the time (outside of the predefined non-inferiority margin of 55% - Men had a 63.5% probability of identifying the ChatGPT answer, higher than women (57.8%, p=0.047) - Those who had used ChatGPT before were more likely to correctly identify the AI answer compared to those who had not (67.4% Vs 57.6%, OR 1.52, p = 0.003) |
| 10 | Evaluate ChatGPT's responses to common patient diabetes-related questions | ChatGPT-3.5  March, 2023 | - 25 FAQ on Diabetes - 5 reviewers evaluated the answers against expert opinion | - Accuracy (“appropriate” or “inappropriate”) | - 19 responses (76%) were deemed appropriate by consensus, while 6 (24%) were considered inappropriate - The responses to questions often included more information than was needed and included sometimes subtle inaccuracies (e.g., about the inheritability of diabetes) - 84% of the responses included a sentence stating the importance of discussing with a health care provider |
| 12 | Evaluate ChatGPT’s responses for assessing obesity questions  in diabetics | ChatGPT-3.5  April, 2023 | - 20 questions on Obesity - 2 reviewers compared answers against American Diabetes Association and American Association of Clinical Endocrinology Guidelines | - Evaluation based on four categories: compatible, compatible but insufficient, partially incompatible, and incompatible with guidelines. | - All responses in the general assessment section were compatible with guidelines - 4 out of 6 nutrition and physical activity responses were compatible with the guidelines, one was insufficient, and one was deemed incompatible - 2 out of 5 pharmacotherapy responses were accurate but incomplete, missing newer drugs such as semaglutide. - ChatGPT struggled to distinguish between medical devices and metabolic surgery, with 2 out of 5 responses partially incompatible with guidelines |
| 30 | Evaluate ChatGPT's responses to exercise-related questions for patients with type 2 diabetes | ChatGPT-4  November, 2023 | - 14 FAQ on exercise for managing type 2 diabetes - 2 reviewers compared ChatGPT answers with American Diabetes Association and American College of Sports Medicine guidelines | - Validity, safety and utility (four-point Likert scale) | - ChatGPT provided generally accurate, safe, and useful responses - 71.4% of responses were rated as completely accurate and 28.6% were rated as accurate but incomplete (frequency and duration of exercise, pre-exercise evaluation, and precautions for diabetic retinopathy) - All responses scored 4/4 for safety and usefulness |
| 32 | Evaluate a DeepDR-LLM system, that combines a large language model and deep learning model for diabetic retinopathy screening and diabetes management ^a^ | DeepDR-LLM  (proprietary integrated image-language system; the LLM was fine-tuned from LLaMA)  April to July, 2023 | - Two-arm prospective study - 12 PCP used the system in a real word setting (unassisted Vs DeepDR-LLM assisted) - 785 patients with diabetes and gradable fundus images were evaluated | - Patients adherence to diabetes management recommendations - DR screening and tertiary referral | Patients evaluated by a DeepDR-LLM assisted PCP had:   - Enhanced self-management behaviors in newly diagnosed diabetes (at 4 weeks increased consumption of fresh fruits, decreased consumption of starchy vegetables, more frequent blood glucose monitoring and better adherence to antidiabetic medication; all p<0.05); - Earlier referral to an ophthalmologist if DR was present (4 days Vs 7 days, p <0.001); - PCP reported high satisfaction and overall satisfaction score of 4.50 out of 5 |
| 33 | Evaluate ChatGPT responses in nutritional management for Type 2 Diabetes and MetS | ChatGPT-3.5-turbo  October, 2023 | - 63 questions on nutrition management for diabetes and MetS patients - 2 reviewers compared ChatGPT answers with Academy of Nutrition and Dietetics guidelines | - Accuracy and clarity (four-point Likert scale) | - ChatGPT clarity was rated as good or excellent, but significant gaps in accuracy for critical nutrition advice were identified (recommendations for energy intake, weight management, specific nutrient guidance, such as fiber and omega-3 fatty). - ChatGPT menus deviated from the specified caloric intake and failed to meet several dietary recommendations |

^a^ There were 4 experiments in this publication; the first 3 are on the technical system development and validation and are not reported on this analysis

^b^ For the question “can diabetes be ruled out if fasting blood sugar is normal?", ChatGPT incorrectly used the range 70–100 mg/dL; ChatGPT’s also failed to include the possibility of diabetes remission through surgical treatment in obese patients

FAQ, frequently asked questions; Vs, versus; OR, odds ratio; PCP, Primary Care Physicians; DR, diabetic retinopathy; MetS, metabolic syndrome

**Table S4.** Summary of objectives, methods and key findings of publications evaluating LLMs on other cardiovascular risk factors

| Study ID | Objective | LLM Type & Evaluation Date | Intervention | Outcome Measures | Key findings |
| --- | --- | --- | --- | --- | --- |
| 1 | Assess CVD prevention recommendations provided by ChatGPT | ChatGPT-3.5  December, 2022 | - 25 questions related to basic CVD prevention - 3 reviewers evaluated the answers against expert opinion | - Consistency on repeated prompts (3 runs) - Accuracy ("appropriate" "inappropriate" or "unreliable") | - 21 out of 25 responses (84%) were rated as appropriate - 4 responses (16%) were inappropriate, primarily due to potential misinformation ^a^ - No responses were rated as unreliable - Answers were consistent across multiple prompts |
| 13 | Evaluate ChatGPT's responses to lifestyle-related diseases | ChatGPT-3.5  July, 2023 | - 20 lifestyle-related disease/disorder case vignettes, each with four specific questions (including obesity, diabetes, CVD, and mental health) - 2 reviewers evaluated the answers against expert opinion | - Accuracy (three-point Likert scale) - Adequacy and guidance - Readability (Flesch-Kincaid Reading Ease Score (FKES) and Flesch-Kincaid Grade Level) - Sentiment (classified as positive, negative, or neutral) | - Accuracy score was 1.83±0.37 out of 2, with most responses considered accurate; there were no inaccurate responses - Guidance score was 1.9±0.21, indicating that most responses provided an actionable advice - Flesch-Kincaid Grade Level was 14.37±0.85 (high school to college level readers) - Flesch Reading Ease Score was 27.8±5.74 ("difficult to read" text) - Responses were rated as having a natural tone in 11 cases and a positive tone in 9 cases; none had a negative tone |
| 15 | Assess individualized exercise recommendations generated by ChatGPT for various clinical populations | ChatGPT-3.5  March, 2023 | - Provide individualized exercise recommendations for 26 clinical populations, including patients with cardiovascular disease, diabetes, hypertension, and other chronic conditions. - 2 reviewers compared ChatGPT answers with American College of Sports Medicine guidelines and Expert Opinion | - Comprehensiveness (presence or absence of content provided for each of 10 prespecified categories) - Accuracy (“concordant” or “discordant”) - Readability (Flesch-Kincaid formula and Flesch reading ease score) - Qualitative analysis with thematic mapping approach | - Interrater reliability (Cohen κ coefficient was calculated to be 1.0, indicating perfect agreement between coders) - Exercise recommendations were 41.2% comprehensive, with significant gaps in critical areas such as frequency, intensity, and time; no populations or categories were fully comprehensive - Only 8% of recommendations provided a reference to the primary literature - Recommendations were 90.7% accurate overall ^b^ - Average Flesch-Kincaid was 13.7 (college level); Flesch reading ease score was 31.1, ("difficult to read" text) - Qualitative Analysis showed recurring themes, potential bias and discrimination ^c^ |
| 20 | Evaluate exercise prescriptions generated by GPT-4 for patients with diverse health conditions | ChatGPT-4  June, 2023 | - Five hypothetical patients with varied health conditions (hypertension, osteoarthritis, anxiety, diabetes, asthma) - ChatGPT was tasked with creating a 30-day exercise program using FITT principle, rate of perceived exertion and safety measures - Program evaluated by expert opinion | - Accuracy and completeness (evaluated with a rigorous three-stage refinement process, including sharing a draft, iterative refinement and final review) | - GPT-4 generated safe, conservative exercise programs emphasizing moderate-intensity workouts. - Programs lacked precision in tailoring exercise to individual needs - ChatGPT tended to overemphasize safety, often at the expense of training progression and intensity increases - Programs were technically sound, but they fell short in areas requiring higher customization (e.g., intensity progression, integration of dietary advice, real-time monitoring) |
| 27 | Compare ChatGPT versions 3.5 and 4.0 when answering FAQ on hyperlipidemia | ChatGPT-3.5  ChatGPT-4  May, 2024 | - 25 FAQ on hyperlipidemia - Prompted using 3 formats "no prompt, patient-friendly prompt, physician-level prompt - Answers compared to Cleveland Clinic's official FAQs responses | - Correctness ("correct", "partially correct" or "incorrect") - Readability (Flesch-Kincaid grade level) | - ChatGPT 4.0 had a higher percentage of correct responses (74.67%) compared to ChatGPT 3.5 (69.33%) - Both versions provided reliable information, with incorrect responses being rare (5% or less) - ChatGPT 4.0 offered more concise and readable responses (FK score 15.45 with ChatGPT 3.5 Vs 13.85 with ChatGPT 4.0) |

^a^ Examples of inappropriate answers: exercise recommendations that could be harmful for specific patient populations; outdated or incorrect medication advice; interpretation of a low-density lipoprotein cholesterol level of 200 mg/dL lacked relevant details, including familial hypercholesterolemia and genetic considerations; responses about inclisiran suggested that it is commercially unavailable

^b^ 53% of inaccuracies were related to preparticipation health screening, where ChatGPT overemphasized the need for medical clearance in low-risk populations; hypertension had the greatest number of misinformation counts

^c^ Several recurring themes emerged among the total sample, including liability and safety, preference for aerobic exercise, and inconsistencies in the terminology used for exercise professionals; importantly AI-generated output showed potential bias and discrimination against certain age-based populations and individuals with disabilities

CVD, cardiovascular disease; FITT, frequency, intensity, time, type; FAQ, frequently asked questions

**Table S5.** Summary of objectives, methods and key findings of publications evaluating LLMs on Atrial Fibrillation

| Study ID | Objective | LLM Type & Evaluation Date^a^ | Intervention | Outcome Measures | Key findings |
| --- | --- | --- | --- | --- | --- |
| 8 | Compare ChatGPT and Bing AI for patient and clinician inquiries on AF | ChatGPT-3.5  Bing Chat (GPT-4) | - 36 AF-related questions (18 patient questions tested on ChatGPT, and 18 clinical questions tested on both models) - 3 reviewers evaluated the answers against expert opinion | - Appropriateness categorized as “appropriate” or “inappropriate” - Clinician questions required evidence-based references and were evaluated on both text and reference accuracy | - ChatGPT provided appropriate responses for 83.3% of patients’ questions, with highest appropriateness in treatment (100%) and medication-related (100%) questions but lower for lifestyle (71.4%) - For clinical questions, ChatGPT and Bing AI showed lower appropriateness, with ChatGPT providing correct responses in 33.3% for text accuracy and 55.5% for reference accuracy; Bing AI performed better in response accuracy (66.6%) but was comparable in reference accuracy (50%); ChatGPT produced fabricated references in 2 clinician-related cases - Reviewer agreement was high, with Cohen’s Kappa ranging from 0.77 to 1 |
| 14 | Evaluate responses by different chat-based AI models (Google Bard, Bing Chat, and ChatGPT Plus) regarding AF and implantable cardiac devices | Google Bard, Bing Chat (GPT-4), ChatGPT-4.0  May to October, 2023 | - 50 patient-centered questions (25 on AF and 25 on implantable cardiac devices) - 3 reviewers evaluated the answers against expert opinion | - Appropriateness to guidelines and clinical practice - Comprehensibility - Readability (word count and Flesch Reading Ease score) - Presence of missing content and confabulation | - For AF questions appropriateness for ChatGPT was 84%, Bing Chat 60% and Google Bard 52% - For implantable cardiac devices questions, appropriateness for ChatGPT was 88%, Bing Chat 72% and Google Bard 16% - 24%-60% of AF questions lacked information and 52-92% of implantable cardiac devices lacked information (being lowest for ChatGPT in both set of questions) - Confabulation appeared in a minority of responses (12% in Google Bard for AF) - Comprehensibility was high in all models (88-100%) - ChatGPT had the highest word count and lowest Flesch Reading Ease score; Google Bard showed the better readability |
| 29 | Evaluate ChatGPT responses on AF patient education | ChatGPT-3.5 | - 16 FAQ on AF - Four prompt formats was used ("no prompt, patient-friendly prompt, physician-level prompt, and statistics and references prompting) - Responses compared to American Heart Association patient materials | - Responses scored as "incorrect", "partially correct" and "correct" or "perfect" - The Flesch-Kincaid grade level | - Majority of ChatGPT's responses were correct (85.9% correct and 4.7% perfect); 1.6% of responses were incorrect, and 7.8% partially correct - No significant difference in responses (correct or perfect) across different prompts (p = 0.350) - ChatGPT provided references in only three (4,7%) of responses - Average Flesch-Kincaid (FK) score was 14.66 (collegiate reading level) |
| 31 | Evaluate ChatGPT's responses to common questions about AF | ChatGPT-3.5  November, 2023 | - 20 AF-related questions - 30 reviewers evaluated the answers against expert opinion | - Accuracy and clarity (four-point Likert scale) | - 600 reviews were performed - 55.5% of answers were rated either "excellent" or "very good”; 7.7% were rated poor (7.7%) - 66.7% of physicians believe that ChatGPT is as a reliable source of information for patients |
| 35 | Compare different LLMs in answering AF related questions | ChatGPT-4 and Gemini-1.0  June, 2023 and January, 2024 for ChatGPT-4  January, 2024 for Gemini | - 20 patient-centered questions and 20 physician-centered questions - Answers evaluated against expert opinion | - Accuracy ("Accurate", "Inaccurate" and "Incomplete") - LLM with the most appropriate answer - Readability (Flesch-Kincaid grade level) | - ChatGPT-4 2024 was the best model (patient questions were 90% accurate and 10% accurate but incomplete; physician questions were 55% accurate, 35% accurate but incomplete and 10% inaccurate) - Gemini showed lower overall accuracy than ChatGPT-4 (33 Vs 73%, p<0.01) - ChatGPT-4 accuracy improved over time (45% in 2023 Vs 73% in 2024) - All models showed greater performance in answering patient versus physician questions (p<0.05) - Flesch-Kincaid grade levels of 11.8 for ChatGPT and 12.7 for Gemini (college-level) |

^a^ If date not reported by authors was left in blank

AF, Atrial Fibrillation; FAQ, frequently asked questions

**Table S6.** Summary of objectives, methods and key findings of publications evaluating LLMs on Heart Failure

| Study ID | Objective | LLM Type & Evaluation Date^a^ | Intervention | Outcome Measures | Key findings |
| --- | --- | --- | --- | --- | --- |
| 19 | Evaluate ChatGPT’s responses to FAQ on HF | ChatGPT-3.5 | - 47 FAQ on HF - 2 reviewers evaluated the answers against heartfailurematters.org | - Accuracy and consistency | - ChatGPT provided correct and comprehensive answers for 41 out of 47 questions (87%) - 5 answers were correct but incomplete and 1 answer had inaccurate information - Responses were consistent across repeated prompts, showing high reproducibility |
| 24 | Evaluate the potential of LLM-based AI chat platforms in answering patients questions on HF | ChatGPT-3.5 and Google Bard  June, 2023 | - 30 FAQ on HF - 2 reviewers evaluated the answers against AHA/ACC/HFSA guidelines | - Accuracy (“adequate”, “incomplete” or “inaccurate”) - Consistency (“consistent” or “not consistent”) | - ChatGPT-3.5 provided 90% accurate answers (27/30), while Bard provided 56% (17/30) - ChatGPT responses were consistent 93% of the time, while Bard did not demonstrate significant variability - Both platforms performed adequately for most questions, but struggled with newer guideline-based management strategies - Bard occasionally hallucinated references or underplayed important risks (e.g., pregnancy in HF) |
| 28 | Evaluate ChatGPT's responses to FAQ on HF | ChatGPT-3.5 and ChatGPT-4 | - 107 FAQ on HF - 2 reviewers evaluated the answers based on expert opinion | - Accuracy ("comprehensive", "correct but inadequate", "some correct and some incorrect" and "completely incorrect") - Reproducibility | - GPT-4 outperformed GPT-3.5, providing 100% correct responses, with 83.2% graded as comprehensive, compared to 98.1% correct and 78.5% comprehensive for GPT-3.5 - Both models demonstrated high reproducibility (100% for GPT-4 and 98% for GPT-3.5) - GPT-4 provided more detailed and accurate responses, particularly in management-related questions - GPT-3.5 had rare responses (1,9%) with incorrect information; GPT-4 did not gave any incorrect information |
| 34 | Evaluate ChatGPT's responses to FAQ on HF | ChatGPT-3.5  November, 2023 | - 12 FAQ on HF - Answers reviewed against AHA/ACC/HFSA educational materials | - Readability scores - Actionability scores, - Educational level - User engagement | - ChatGPT's responses were found to be longer and more challenging to read, written at a higher educational level - Despite a competitive readability score of 75%, ChatGPT's actionability score was the lowest at 67% - ChatGPT's output included a high percentage of difficult words not found in the Dale-Chall word list |

^a^ If date not reported by authors was left in blank

FAQ, frequently asked questions; HF, Heart Failure; AHA/ACC/HFSA, American Heart Association / American College of Cardiology / Heart Failure Society of America

**Table S7.** Summary of objectives, methods and key findings of publications evaluating LLMs on other cardiovascular conditions

| Study ID | Objective | LLM Type & Evaluation Date^a^ | Intervention | Outcome Measures | Key findings |
| --- | --- | --- | --- | --- | --- |
| 5 | Evaluate Bing Chat performance in providing assistance for common cardiovascular conditions | Bing Chat (GPT-4)  February, 2023 | - 14 simulated patients with cardiovascular-related health conditions using a freestyle-like conversation - 2 reviewers evaluated the answers based on expert opinion | Evaluation based on the following:   - Appropriate final decision Thorough anamnesis Responses clear and easy to understand | - Bing Chat provided appropriate and safe final advice in all 14 cases (100% of cases) - An appropriate anamnesis was found in 10 out of 14 cases (71%). In four cases, critical symptoms were not questioned - 93% of the chatbot's responses were rated as clear and easy to understand |
| 7 | Evaluate ChatGPT-4.0 performance in providing prediagnosis and treatment plans for cardiac clinical cases | ChatGPT-4 | - 20 cardiology clinical cases (developed by experienced cardiologists) - 18 reviewers evaluated the answers based on expert opinion | - Agreement with diagnosis and quality of management plan | - ChatGPT-4.0 had a high physician adherence rate to its diagnoses (median 5.00, IQR 1) - ChatGPT-4.0's management plan received a median score of 4 (IQR 1), indicating a good quality of response as perceived by the physicians. - ChatGPT performance was consistent regardless of case complexity |
| 9 | Evaluate the quality of first aid advice provided by Bing Chat for heart attack queries in three countries | Bing Chat (GPT-4)  May, 2023 | - A “heart attack what to do” query (simulating users seeking first aid advice) was repeated 20 times in 3 countries (Gambia, India, and USA) - 2 reviewers evaluated the answers based on the checklist from International First Aid, Resuscitation, and Education Guidelines 2020 | - Compliance with checklist - Length (number of sentences) and readability (Flesch-Kincaid Grade Level) | - The chatbot responses had similar length - The readability differed, being lower for the Gambia and the USA (requiring approximately 12th education grade to understand the text) than for India (requiring approximately 10th grade); p=0.008 - Full congruence (completely satisfied) with checklist items was low, ranging from 7.3% in India, 8.6% in Gambia and 16.8% in the USA - Omissions and inaccuracies were frequent; reproducibility was low and did not adapt to geographical variability ^b^ |
| 16 | Evaluate ChatGPT's responses to FAQ on ACS | ChatGPT-3.5 | - 72 FAQ on ACS (patient and clinical questions) - 2 reviewers evaluated the answers based on expert opinion ESC guidelines | - Accuracy and proficiency using the Global Quality Score (ranges from 1, lowest quality, to 5, highest quality) - Reproducibility | - ChatGPT achieved high accuracy, with 65 (90.3%) of its responses scoring GQS 5 (highest accuracy and proficiency). None of the responses scored GQS 1 (lowest). - Highest accuracy was observed in the prevention and lifestyle section, where 19 (95%) responses scored GQS 5 and 1 (5%) response scored GQS 4 - ChatGPT performed less effectively in the treatment and management section - Reproducibility was high, with 94.4% reproducibility for FAQs |
| 22 | Ability of ChatGPT to support clinical decision in severe AS | ChatGPT-4 | - 150 patients with severe AS discussed in Heart Team meetings - ChatGPT was asked treatment recommendations (TAVI, SAVR, or medical management) based on clinical vignettes containing 14 key variables | - Agreement rate between ChatGPT and Heart Team decisions - Agreement rate between Heart Team and guideline-derived decision trees (ESC and AHA) | - ChatGPT’s decisions agreed with Heart Team decisions 77% of the time overall. The agreement rate was 90% for TAVI, 65% for SAVR, and 65% for medical treatment. - 35 patients were misclassified (TAVI instead of SAVR for 21 patients and SAVR or medical treatment instead of TAVI for 7 patients) - The ESC guideline-based decision tree agreed with HT decisions 73% of the time, while the AHA guideline-derived tree showed lower accuracy at 43% |
| 23 | Assess accuracy of ChatGPT to Advanced Cardiovascular Life Support guidelines in managing cardiac arrest and bradycardia | ChatGPT-4  May to August, 2023 | - 2 simulated clinical scenarios (cardiac arrest and bradycardia management); each scenario was simulated 20 times using a step-by-step decision-making - 3 reviewers evaluated the answers based AHA ACLS guidelines | - Accuracy for each individual Step - Accuracy for each algorithm over 20 simulations - For each simulation step, ChatGPT was scored for correctness (1 point) or incorrectness (0 points) | - ChatGPT’s median accuracy for each step was 85% (IQR 40%-100%) for cardiac arrest and 30% (IQR 13%-81%) for bradycardia - ChatGPT median accuracy for Cardiac Arrest over 20 Simulations was 69% (IQR 67%-74%) and for Bradycardia was 42% (IQR 33%-50%) - ChatGPT’s outputs varied despite consistent input^c^; a lack of step-by-step guided consistency was found |
| 26 | Evaluate two LLMs in responding to rehabilitation concerns from stroke survivor patients and caregivers | ChatGPT and Google Bard  February, 2024 | - 10 questions curated from stroke patients and caregivers - 3 reviewers evaluated the answers based on expert opinion | - Accuracy, safety, relevance, and readability (three-point Likert scale) | - ChatGPT received 79 satisfactory grades (65.8%) and Google Bard received 91 (75.8%) - Both chatbots demonstrated good readability (90% in ChatGPT and 86.7% in Google Bard) - Both chatbots had hallucinations providing non-existent resources (26.7% of answers were borderline or unsatisfactory on both models); and both performed poorly in recognizing emotional or mental health risks (for safety 53.3% of answers were borderline or unsatisfactory with ChatGPT and 36.6% with Google Bard) - Inter-rater agreement among clinicians was low |

^a^ If date not reported by authors was left in blank

^b^ Common omissions included crucial life-saving advice, such as starting CPR for an unresponsive person, stopping physical activity, or calling for EMS; in many cases, the chatbot provided incorrect instructions, such as referencing the incorrect emergency number or omitting the recommended dosage for aspirin (150-300 mg); superfluous directives, like instructing users to open windows or check the pulse, were also noted; responses were inconsistent across repeated queries; the advice given varied in completeness and accuracy, with some suggestions deviating significantly from first aid guidelines; the chatbot did not sufficiently adapt to different regions, providing instructions such as asking for automatic external defibrillators in regions where they are unlikely to be available (e.g., the Gambia)

^c^ The same actions were persistently missed (failure to establish IV/IO access, obtain ECGs), repetitive overemphasis hindered guidance (repeated “check rhythm”), and erroneous medication information (incorrect atropine dosages) was presented

FAQ, frequently asked questions; ACS, acute coronary syndromes; ESC, European Society of Cardiology; AS, aortic stenosis; TAVI, transcatheter aortic valve implantation; SAVR, surgical aortic valve replacement; AHA, American Heart Association; ACLS, advanced cardiovascular life support

**Table S8: Keywords used in article search (medical LLMs only)**

A comprehensive and automated search strategy was performed in Pubmed, using terms related to Large Language Models specifically developed for medical domains and Cardiovascular Disease, on November 10, 2024, using the following keywords.

| **#** | **Search Keywords (In Title)** | **PubMed**  **Results** |
| --- | --- | --- |
| 1 | “Med-PaLM” OR "BioMistral" OR "BioBERT" OR "ClinicalBERT" | 6 |
| 2 | “Cardiology” OR "Cardiovascular Disease" OR "Heart Disease*" OR “Heart” OR "Cardiac Disease" OR "Coronary Artery Disease" OR “Angina” OR “Acute Coronary Syndrome*” OR "Myocardial Infarction" OR "Heart Failure" OR "Atrial Fibrillation" OR "Stroke" OR "Peripheral Artery Disease" OR "Cardiomyopathy" OR "Valvular Heart Disease" OR “Heart Valve Disease*” OR "Congenital Heart Disease" OR "Risk Factors" OR "Hypertension" OR "Blood Pressure" OR "Diabetes" OR "Type 1 Diabetes" OR "Type 2 Diabetes" OR "Hyperlipidemia*" OR "Cholesterol” OR “Dyslipidemia" OR "Elevated Lipids" OR "Hypercholesterolemia" OR "Obesity" OR "Overweight" OR "Excess Weight" OR "Smoking" OR "Tobacco Use" OR "Cigarette Smoking" OR "Nicotine" OR "Lifestyle Interventions" OR "Diet" OR "Physical Activity" OR "Physical Inactivity" OR "Exercise" OR "Smoking Cessation" OR "Alcohol" OR "Weight" OR "Behav* Change" OR “Behavior Therapy” OR “Lifestyle” | 2,162,358 |
| 3 | 1 AND 2 | 0 |

**Figure S1: ClinicalTrials.Gov**

Results of a search using keywords on LLMs from supplementary table 1, performed on November 10, 2024


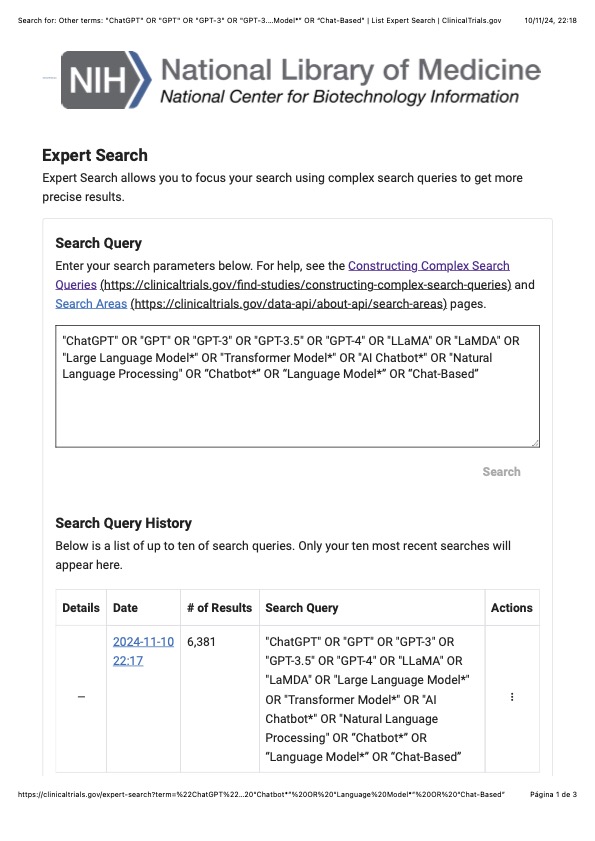

Supplement: ztaf028_Supplementary_Data [file ztaf028_supplementary_data.docx]
